# Deficient Rnf43 potentiates hyperactive Kras‐mediated pancreatic preneoplasia initiation and malignant transformation

**DOI:** 10.1002/ame2.12203

**Published:** 2022-01-22

**Authors:** Xian Zhou, Zhichao Sun, Mengdi Zhang, Xiaoyu Qu, Shuhui Yang, Lianmei Wang, Yanling Jing, Li Li, Weiwei Deng, Fangming Liu, Jin Di, Jie Chen, Jian Wu, Hongbing Zhang

**Affiliations:** ^1^ State Key Laboratory of Medical Molecular Biology Department of Physiology Institute of Basic Medical Sciences and School of Basic Medicine Chinese Academy of Medical Sciences and Peking Union Medical College Beijing China; ^2^ Institute of Cancer Stem Cell Dalian Medical University Dalian China; ^3^ Institute of Chinese Materia Medica China Academy of Chinese Medical Sciences Beijing China; ^4^ Department of Pathology Peking Union Medical College Hospital Chinese Academy of Medical Sciences Beijing China; ^5^ MyGenostics Inc. Beijing China

**Keywords:** intraductal papillary mucinous neoplasms, KRAS, pancreatic ductal adenocarcinoma, RNF43, Wnt

## Abstract

**Background:**

Largely due to incidental detection, asymptomatic pancreatic cystic lesions (PCLs) have become prevalent in recent years. Among them, intraductal papillary mucinous neoplasm (IPMN) infrequently advances to pancreatic ductal adenocarcinoma (PDAC). Conservative surveillance versus surgical intervention is a difficult clinical decision for both caregivers and PCL patients. Because *RNF43* loss‐of‐function mutations and *KRAS* gain‐of‐function mutations concur in a subset of IPMN and PDAC, their biological significance and therapeutic potential should be elucidated.

**Methods:**

Pancreatic *Rnf43* knockout and *Kras* activated mice (*Rnf43* ^−/−^; *Kras^G12D^*) were generated to evaluate their clinical significance in pancreatic pre‐neoplastic initiation and malignant transformation.

**Results:**

Loss of *Rnf43* potentiated the occurrence and severity of IPMN and PDAC in oncogenic *Kras* mice. The Wnt/β‐catenin signaling pathway was activated in pancreatic *Kras^G12D^* and *Rnf43* knockout mice and the PORCN inhibitor LGK974 blocked pancreatic IPMN initiation and progression to PDAC accordingly.

**Conclusions:**

Rnf43 is a tumor suppressor in the prevention of pancreatic malignant transformation. This genetically reconstituted autochthonous pancreatic *Rnf43* ^−/−^; *Kras^G12D^* preclinical cancer model recapitulates the pathological process from pancreatic cyst to cancer in humans and can be treated with inhibitors of Wnt/β‐catenin signaling. Since the presence of *RNF43* and *KRAS* mutations in IPMNs predicts future development of advanced neoplasia from PCLs, patients with these genetic anomalies warrant surveillance, surgery, and/or targeted therapeutics such as Wnt/β‐catenin inhibitors.

## Graphical abstract

Pancreatic *Rnf43* knockout and *Kras* activated mice (*Rnf43^−/−^*; *Kras^G12D^*) develop from pancreatic cysts to pancreatic ductal adenocarcinoma which can be blunted by suppression of Wnt/β‐catenin signaling pathway.

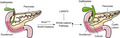

## 1 INTRODUCTION

With a pooled prevalence of 8% in adults, pancreatic cystic lesions (PCLs) are often asymptomatically and incidentally discovered on routine cross‐sectional abdominal imaging.^1^ PCLs are a heterogeneous group of benign tumors and precursor lesions of pancreatic ductal adenocarcinoma (PDAC). PDAC is a major type of pancreatic malignant tumor, with the worst prognosis among cancers.^2^ Even though the malignant potential of PCL is very low, accurate diagnosis and correct assessment of malignant potential are often hard to achieve. Since it is a daunting task to distinguish tumors with or without malignant potentials, perceived dire prognosis of PDAC, evolved from incidentally detected PCLs, often forces both caregivers and patients to choose between major pancreatic resection, with substantial morbidity and mortality, and life‐long surveillance, with associated financial burdens, anxiety, and the risk of missed malignancy.

Intraductal papillary mucinous neoplasm (IPMN) is a major type of PCL, which may progress from low‐grade dysplasia (LGD) to high‐grade dysplasia (HGD), and eventually to PDAC.^3^ Therefore, IPMN is a precursor lesion of PDAC.^3^, ^4^, ^5^ Progression from precancerous lesions to invasive pancreatic cancer may take about 20 years.^6^ It is a curable disease if detected and treated before it proceeds to invasive carcinoma. In clinical practice, LGD can potentially be managed conservatively while HGD and invasive carcinoma would require surgical resection.^7^, ^8^ The surgical indication for these patients is based on the presence of indirect predictors in diagnostic images. Given the lack of information on the natural history of IPMN malignant transformation, whether noninvasive carcinoma or HGD should be operated on or observed remains an unsolved practical issue. Therefore, analysis of these precancerous lesions is crucial to understand the earliest events of pancreatic tumorigenesis.

Recent studies have shown that the most frequently mutated genes in IPMNs are *KRAS*, *GNAS*, and *RNF43*.^9^, ^10^ We and others identified that the pooled prevalence of *RNF43* inactivating mutation in IPMN was 24% by next generation sequencing assessment.^9^, ^11^, ^12^, ^13^ *RNF43* inactivating mutation and *KRAS* activating mutation coexist in a subset of IPMN tumor tissues.^10^, ^11^, ^12^ *RNF43* is also altered in 7% of invasive PDACs with or without obvious IPMN.^14^

RNF43 is a member of the RING finger protein family,^15^ and an E3 ubiquitin ligase.^13^, ^16^ It exerts negative feedback regulation of the Wnt/β‐catenin signaling pathway by (a) mediating the ubiquitination and degradation of the Wnt receptor complex component Frizzled; and (b) tethering TCF4 to the nuclear membrane for silencing TCF4 transcriptional activity. Loss of RNF43 activates Wnt/β‐catenin signaling pathway.^17^, ^18^, ^19^ To dissect potential interplaying roles and underlying mechanisms of aberrant *RNF43* and *KRAS* in IPMN initiation and malignant transformation, we simulated human IPMN and PDAC in mice by establishing a pancreatic *Kras^G12D^* and *Rnf43* deficient preclinical model. We also developed a noninvasive therapeutic regimen for the unmet needs of this disturbing disease.

## 2 METHODS

### 2.1 Generation of mouse models

All animal experiments were approved by the Animal Care and Use Committee at Peking Union Medical College and were performed in accordance with international guidelines. *Pdx1*‐cre, LSL‐ *Kras* ^G12D^ mice have been described previously.^20^ Conditional *Rnf43* ^LoxP/LoxP^ mice were established through a contract with Beijing Biocytogen. Mice were kept on a C57BL/6 genetic background. LoxP sites were inserted into the introns before exon 7 and after exon 8 of *Rnf43* alleles. To generate pancreatic *Kras* activation and *Rnf43* knockout mice, we crossed *Pdx1*‐cre mice (B6.FVB‐Tg (Ipf1‐cre)1Tuv/Nc), LSL‐*Kras* ^G12D^ mice and *Rnf43* ^LoxP/LoxP^ mice to produce *Pdx1*‐cre; LSL‐*Kras* ^G12D^; *Rnf43* ^LoxP/LoxP^ (*Rnf43* ^−/−^; *Kras* ^G12D^) mice, *Pdx1*‐cre; LSL‐*Kras* ^G12D^ (*Kras* ^G12D^) mice, *Pdx1*‐cre; *Rnf43* ^LoxP/LoxP^ (*Rnf43* ^−/−^) mice, and *Rnf43* ^LoxP/LoxP^ (*Rnf43* ^f/f^) mice. These mice were sacrificed at the ages of 16 days, 1 month, and 6 months. Tissues were fixed in 10% formalin for 24 hours and then paraffin embedded for histological staining, or frozen in liquid nitrogen and stored at −80°C. Genotyping was conducted using tails of 10‐ to 14‐day‐old mice. DNA was extracted from tails in lysis buffer (100 μl DirectPCR Lysis Regent (#102‐T, VIAGEN BIOTECH), 2 μl Protein Kinase (#E195, Ameresco) (at 56°C overnight and 85°C for 45 min. PCR was performed according to standard procedures.

The primers used were as follows: Flp forward: 5′‐TTCGAATCATCGGAAGAAGC‐3′; Flp reverse: 5′‐ACTCCGTTAGGCCCTTCATT‐3′; Neo forward: 5′‐GCACCGCTGAGCAATGGAAG‐3′; Neo reverse: 5′‐AGCCGATTGTCTGTTGTGCC‐3′; Rnf43‐A1‐loxp forward: 5′‐ACTAGGCACACCCCAGGTGC‐3′; Rnf43‐A2‐loxp reverse: 5′‐ AGCTCTTGCACAGCTGTGACC‐3′; Rnf43‐Frt forward: 5′‐GAAGGTTG TCCATACTGAAACCATCTTTG‐3′; Pdx1‐Cre forward: 5′‐TGGGCGGC ATGGTGCAAGTT‐3′; Pdx1‐Cre reverse: 5′‐CGGTGCTAACCAGCGTTTTC‐3′; Rnf43‐E7 forward: 5′‐GATCCTCTTCAGCAGCGGAC‐3′; Rnf43‐E7 reverse: 5′‐CGGTGCTAACCAGCGTTTTC‐3′; Kras‐8272: 5′‐GTCGACAAGCTCATGCGGG‐3′; Kras‐8273: 5′‐CGCAGACTGTAGAGCAGCG‐3′; Kras‐8274: 5′‐CCATGGCTTGAGTAAGTCTGC‐3′.

### 2.2 Histological and immunohistochemical analyses

After fixing in 10% formalin for more than 24 hours, tissues were embedded in paraffin and cut to 4‐μm sections. H&E and immunohistochemical staining was performed according to standard procedures. Antibodies utilized are listed in Table 1. Photos were taken with Leica DMi8 microscope, with identical adjustments and exposure times between genotypes and treatment groups. Immunohistochemical staining images were captured using CaseViewer software (3DHISTECH) and quantified with Image‐Pro Plus 6.0 software (Media Cybernetics).

**TABLE 1:** Lists of antibodies used in the immunoblotting and immunohistochemical analyses

Antibody	Spies	Vendors	Catalog	Immunoblotting	Immunohistochemical analyses
RasG12D	Rabbit	Cell Signaling Technology	14429s	1:1000	
GAPDH	Mouse	ABclonal	AC033	1:1000	
Gl‐ Syn	Mouse	Santa Cruz Biotechnology	sc‐74430		1:100
Cytokeratin 19	Mouse	Abcam	ab133496		1:2000
β‐catenin	Rabbit	Cell Signaling Technology	9587		1:800
HRP conjugated anti‐rabbit IgG (H + L)	Goat	Servicebio	GB23303		1:200
HRP conjugated anti‐mouse IgG (H + L)	Goat	Servicebio	GB23301		1:200
IRDye 800CW anti‐rabbit IgG	Goat	LI‐COR	926‐32211	1:10000	
IRDye 800CW anti‐mouse IgG	Goat	LI‐COR	926‐32210	1:10000	

### 2.3 Membrane bound O‐acyl transferase porcupine (PORCN) inhibitor LGK974 treatment

LGK974 (#S7143, Selleck) was formulated in 0.5% MC/0.5% Tween‐80 (MC: low viscosity, #BB12BA0026, BBI Life Sciences; Tween‐80: #CB01BA0035; BBI Life Sciences). *Rnf43* ^−/−^; *Kras* ^G12D^ mice were randomly assigned to control (21 mice in total, 12 ♂ 9 ♀) or treatment groups (22 mice in total, 14 ♂ 8 ♀). Treatment started at 20 days after birth. LGK974 was administered by oral gavage at a dosage of 5 mg/kg animal body weight, twice daily, for 14 consecutive days. Controls were given corresponding volumes of 0.5% MC/0.5% Tween‐80. Body weight was monitored twice daily for 14 treatment days. Mice were observed daily for survival and sacrificed at the age of 6 months. Tissues were fixed in 10% formalin for 24 hours and then paraffin embedded for histological and immunostaining.

### 2.4 Immunoblotting

Pancreatic tissues were ground in liquid nitrogen and then sonicated (power 20%, 5 seconds on, 5 seconds off, total time 5 minutes) in lysis buffer (2% SDS, 10% glycerol, 10 mM Tris (pH 6.8) and 100 mM DTT). The tissue suspension was then centrifuged for 10 minutes (4°C, 12,000 rpm). The supernatant was removed and boiled for 10 minutes. Protein lysates were resolved on NuPAGE™ 4%‐12% Bis‐Tris Mini Protein Gel (#NP0336BOX; Invitrogen) and transferred to 0.45 μm nitrocellulose membranes (#HATF00010; Merck). Protein samples were normalized to GAPDH. Antibodies utilized are listed in Table 1. Imaging was taken using LI‐COR Odyssey CLx.

### 2.5 cBioPortal analysis of gene mutations in human pancreatic adenocarcinoma

Pancreatic cancer genomics data deposited in ICGC, QCMG, UTSW and TCGA database were analyzed using the cBioPortal for Cancer Genomics (http://cbioportal.org) for genetic alterations of KRAS, TP53 and RNF43.

### 2.6 Statistical analysis

The Kaplan‐Meier log‐rank test was used for analysis of mouse survival and body weight differentiation using GraphPad Prism software (Version 7). All quantitative data are reported as means ± SD unless otherwise indicated in the figure legends. A *p*‐value of <0.05 was considered as significant.

## 3 RESULTS

### 3.1 Pancreatic *Rnf43*‐deficient mice have enlarged pancreata

**FIGURE 1:**
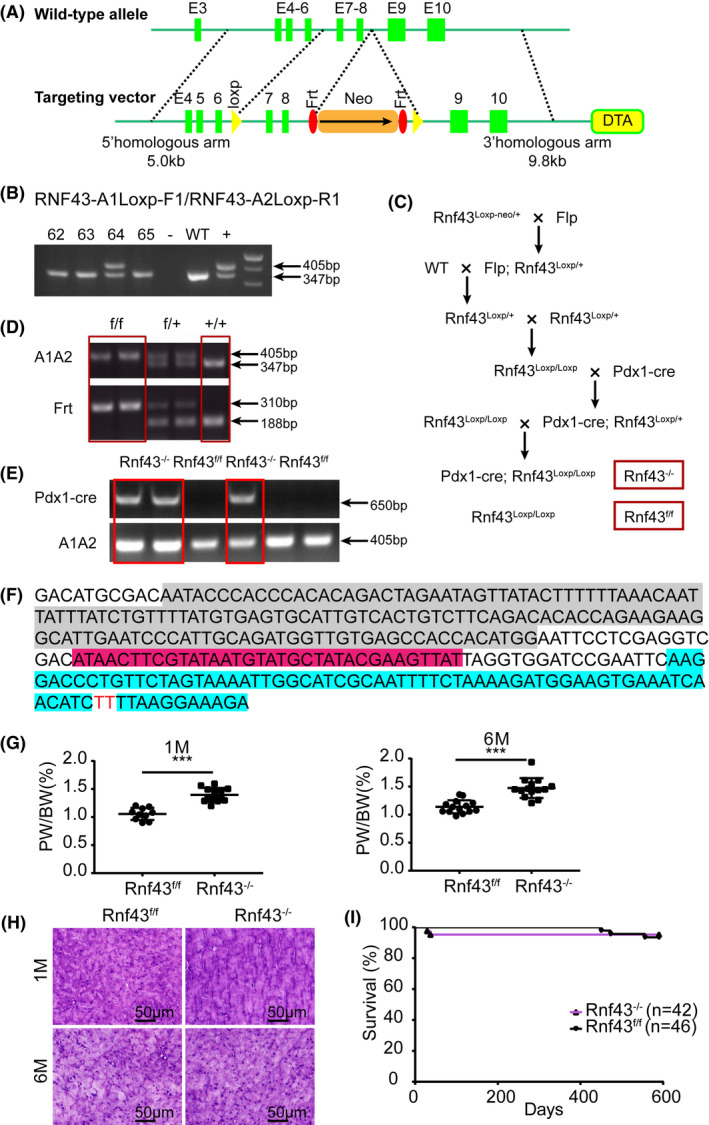
Pancreatic *Rnf43* ^−/−^ mice have larger pancreata. A, Targeted allele of *Rnf43* conditional knockout mice. B, PCR analysis of mouse tail genomic DNA. #64 is *Rnf43* ^LoxP−neo/+^ mouse; #62,63 and 65 are *Rnf43* ^+/+^ mice; − is blank control; WT is negative control; + is positive cloned genomic DNA. C, Cross‐mating schedule for generating pancreatic *Rnf43* knockout mice (*Rnf43* ^−/−^) and their control littermates (*Rnf43* ^f/f^). D, Identification of *Rnf43* ^LoxP/LoxP^ mice by genomic DNA PCR. f/f: *Rnf43* ^LoxP/LoxP^; f/+: *Rnf43* ^LoxP/+^; +/+: *Rnf43* ^+/+^. E, Identification of *Pdx1*‐cre; *Rnf43* ^LoxP/LoxP^ mice by genomic DNA PCR. F, Genomic DNA sequencing of *Rnf43* ^−/−^ mouse. Gray: 5′ homologous arm; rose: LoxP sequence; blue: 3′ homologous arm. G, Ratio of pancreas weight to body weight of 1‐month‐old (left) and 6‐month‐old (right) *Rnf43* ^f/f^ and *Rnf43* ^−/−^ mice. Means ± SD. ***p < 0.001. H, H&E staining of pancreata from 1‐month‐old and 6‐month‐old *Rnf43* ^f/f^ and *Rnf43* ^−/−^ mice. Scale bar, 50 μm. I, Kaplan‐Meier curves of *Rnf43* ^−/−^ mice (purple) (n = 42) and *Rnf43* ^f/f^ mice (black) (n = 46)

We and others identified mutated *RNF43* in tumor tissues of IPMN patients.^11^, ^12^ To check whether loss of RNF43 causes IPMN, we generated pancreatic *Rnf43* knockout mice. First, conditional *Rnf43* knockout mice were created by inserting LoxP and neo cassette into the introns flanking exon 7 and exon 8 of *Rnf43* gene (Figure 1A). *Rnf43^LoxP^* ^−^ *^neo^* ^/+^ mouse (#64) was identified by genotyping analysis (Figure 1B). Neo cassette was then removed by crossing *Rnf43 ^LoxP^* ^−^ *^neo^* ^/+^ mice with Flp‐deleter mice (Figure 1C,D). Lastly, *Rnf43* ^f/f^ mice were mated with pancreatic specific Cre (*Pdx1*‐cre) mice to produce pancreatic *Rnf43* knockout mice (*Pdx1*‐cre; *Rnf43^LoxP^* ^/^ *^LoxP^* (*Rnf43* ^−/−^)) (Figure 1C). Knockout of *Rnf43* in the pancreata of mice was detected by genotyping analysis (Figure 1E). DNA sequencing confirmed that exons 7 and 8 were removed from *Rnf43* gene (Figure 1F).

*Rnf43* ^−/−^ and *Rnf43^f^* ^/^ *^f^* mice were viable and born at the expected Mendelian ratio. Pancreas weight of *Rnf43* ^−/−^ mice was heavier than that of *Rnf43^f^* ^/^ *^f^* mice at both 1 month and 6 months after birth (Figure 1G). Histopathological analysis did not reveal obvious alterations in the architecture of the pancreata in *Rnf43* ^−/−^ and *Rnf43^f^* ^/^ *^f^* mice at both time points (Figure 1H). Survival of *Rnf43* ^−/−^ mice was no worse than that of wild type mice (Figure 1I). Even though the loss of *Rnf43* led to bigger pancreata, this was not sufficient to cause tumor.

### 3.2 Pancreatic *Kras^G12D^* and *Rnf43* deficient mice develop intraductal papillary mucinous neoplasms

**FIGURE 2:**
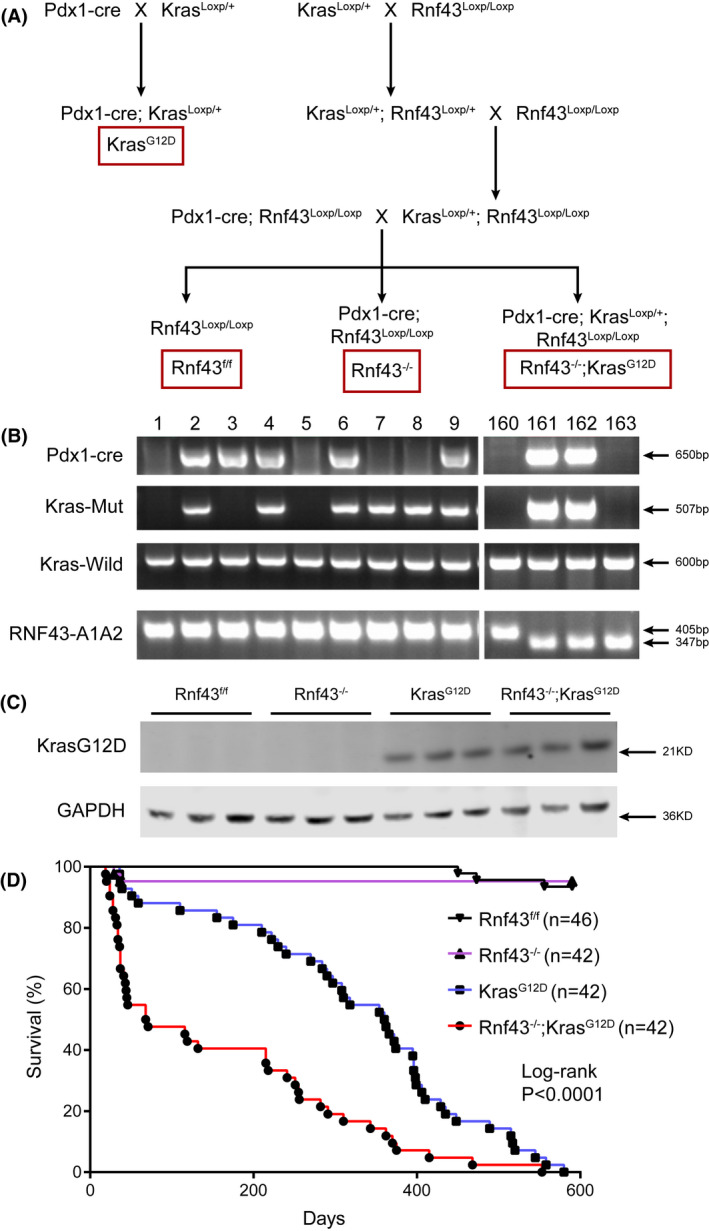
*Rnf43* ^−/−^; *Kras^G12D^* mice have shortened lifespan. A, Cross‐mating schedule for generating pancreatic *Kras* ^G12D^ and *Rnf43* deficient mice. B, PCR genotyping of mouse tail genomic DNA. #2, 4, 6 and 9 are *Rnf43* ^−/−^; *Kras* ^G12D^; #161 and 162 are *Kras* ^G12D^; #3 is *Rnf43* ^−/−^; #1, 5 and 160 are *Rnf43* ^f/f^. C, Immunoblotting of pancreatic lysates from 6‐month‐old *Rnf*43^−/−^; *Kras* ^G12D^ and control mice. D, Kaplan‐Meier curves of *Rnf43* ^−/−^; *Kras* ^G12D^ mice (red) (n = 42) comparing with *Rnf43* ^f/f^ (black) (n = 46), *Rnf43* ^−/−^ (purple) (n = 42) and *Kras* ^G12D^ (blue) (n = 42) mice. Log‐rank *p* < 0.0001

Oncogenic *KRAS* mutations are the earliest driver gene alterations in IPMNs.^21^ Mutations of *KRAS* and *RNF43* often concur in a subset of IPMNs.^11^, ^12^ To decipher the causative relationship between concomitant *RNF43*/*KRAS* mutations and pancreatic carcinogenesis, we generated pancreatic *Rnf43* knockout and *Kras* activation mice (*Rnf43* ^−/−^; *Kras^G12D^*) through cross‐mating of *Pdx1*‐cre mice with *Rnf43^LoxP^* ^/^ *^LoxP^* mice and *Kras^LoxP^* ^/+^ mice (Figure 2A,B). KRAS^G12D^ protein was only detected in the pancreata of *Rnf43* ^−/−^; *Kras^G12D^* and *Pdx1*‐cre; *Kras^LoxP^* ^/+^ (*Kras^G12D^*) mice but not in that of *Rnf43^f^* ^/^ *^f^* and *Rnf43* ^−/−^ mice (Figure 2C). *Rnf43* deficiency could not be verified at the protein level due to lack of an effective antibody against mouse Rnf43. *Rnf43* ^−/−^; *Kras^G12D^* mice had shortened lifespans compared to *Rnf43^f^* ^/^ *^f^*, *Rnf43* ^−/−^, or *Kras^G12D^* mice (Figure 2D).

**FIGURE 3:**
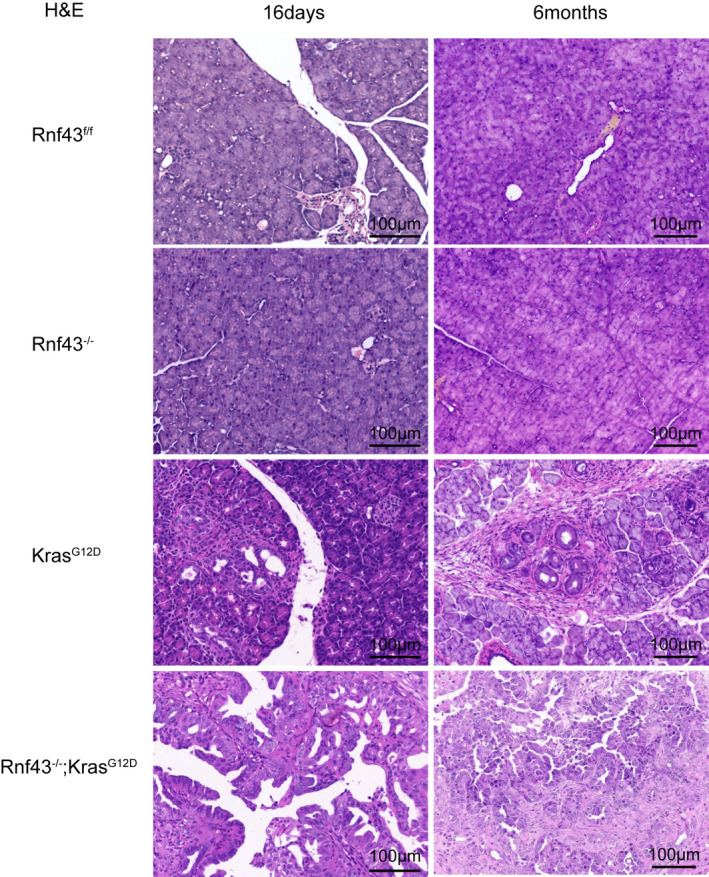
*Rnf43* ^−/−^; *Kras* ^G12D^ mice develop pancreatic intraductal papillary mucinous neoplasms and pancreatic intraepithelial neoplasia. H&E staining (scale bar, 100 μm) of pancreata from 16‐day‐old (left) and 6‐month‐old *Rnf43* ^f/f^, *Kras* ^G12D^ and *Rnf*43^−/−^; *Kras* ^G12D^ mice

Although *Kras^G12D^* mice develop mouse pancreatic intraepithelial neoplasia (PanIN) lesions, these lesions rarely progress to invasive carcinoma within 1 year.^20^ Consistent with this previous report, *Kras^G12D^* mice showed modest proliferation of duct‐epithelial cells with low‐grade atypia that corresponded to acinar‐to‐ductal metaplasia (ADM) by 16 days of age. *Rnf43* ^−/−^; *Kras^G12D^* mice began to develop pancreatic cystic tumors 16 days after birth. Tumors consisted of dilated ducts with prominent proliferation of epithelial cells. Around 6 months after birth, all *Rnf43* ^−/−^; *Kras^G12D^* mice had widespread IPMN and PanINs embedded in fibrotic stroma with almost no normal acinar cells while *Kras^G12D^* mice had duct‐epithelial cell atypia that corresponded to PanINs (Figure 3). These data suggest that deficient *Rnf43* and mutant *Kras* synergistically promote mouse pancreatic tumorigenesis, which mimics the cardinal features of human IPMN.

### 3.3 Pancreatic *Kras^G12D^* and *Rnf43* deficient mice develop invasive and metastatic PDAC

**FIGURE 4:**
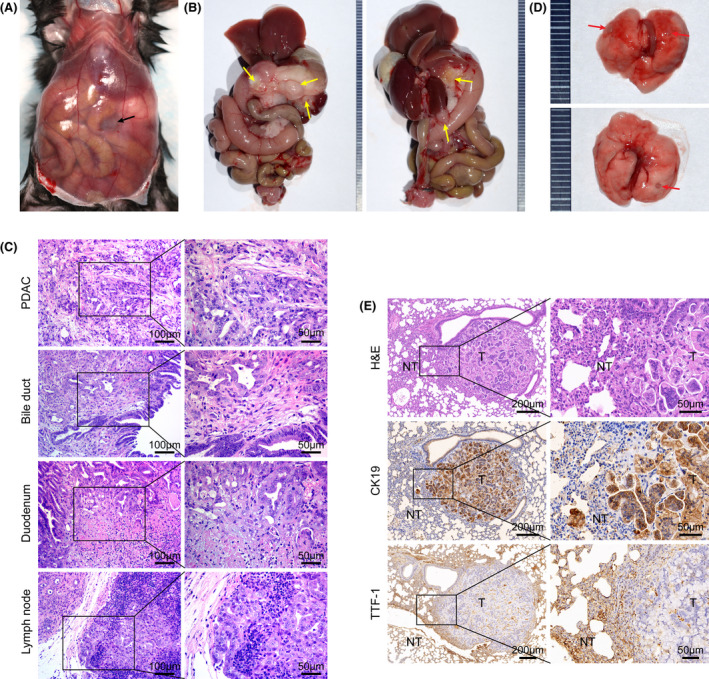
*Rnf43* ^−/−^; *Kras* ^G12D^ mice develop invasive and metastatic pancreatic ductal adenocarcinoma. A, B, Gross abdominal images of a 6.5‐month‐old *Rnf43* ^−/−^; *Kras* ^G12D^ mouse. A, Abdominal distension due to accumulation of ascetic fluid (black arrows). B, PDAC (yellow arrows) in pancreas and its invasion of adjacent organs. C, H&E Staining of invasive PDAC, bile duct, duodenum, and lymph node around the pancreas. Scale bar, left: 100 μm; right: 50 μm. D, Tumor nodules (red arrows) in the lungs of the mouse (A, B). H&E staining, IHC staining of CK19 and TTF‐1 (E) of lung tumor nodules (D). T, tumor; NT, non‐tumor. Scale bar, left, 200 μm; right, 50 μm

At about 6 to 7 months after birth, several *Rnf43* ^−/−^; *Kras^G12D^* mice had abdominal distension owing to the accumulation of malignant ascites (Figure 4A). Large firm tumors in the head of the pancreas were almost invariably seen (Figure 4B). Possible invasion of neighboring structures by pancreatic head tumors resembles the classic features of human pancreatic cancer. Pathologic analysis demonstrated PDAC and its invasion to adjacent bile duct, small bowel, and lymph nodes (Figure 4C). Since the tumor nodules in *Rnf43* ^−/−^; *Kras^G12D^* lungs were positive for duct marker CK19 and negative for pulmonary adenocarcinoma marker thyroid transcription factor 1 (TTF‐1) (Figure 4D,E), they are likely to be metastatic PDAC but not spontaneous lung tumor. Therefore, our data show that deficient *Rnf43* and mutant *Kras* mice develop invasive and metastatic PDAC.

### 3.4 Biliary obstruction in pancreatic *Kras^G12D^* and *Rnf43* deficient mice

**FIGURE 5:**
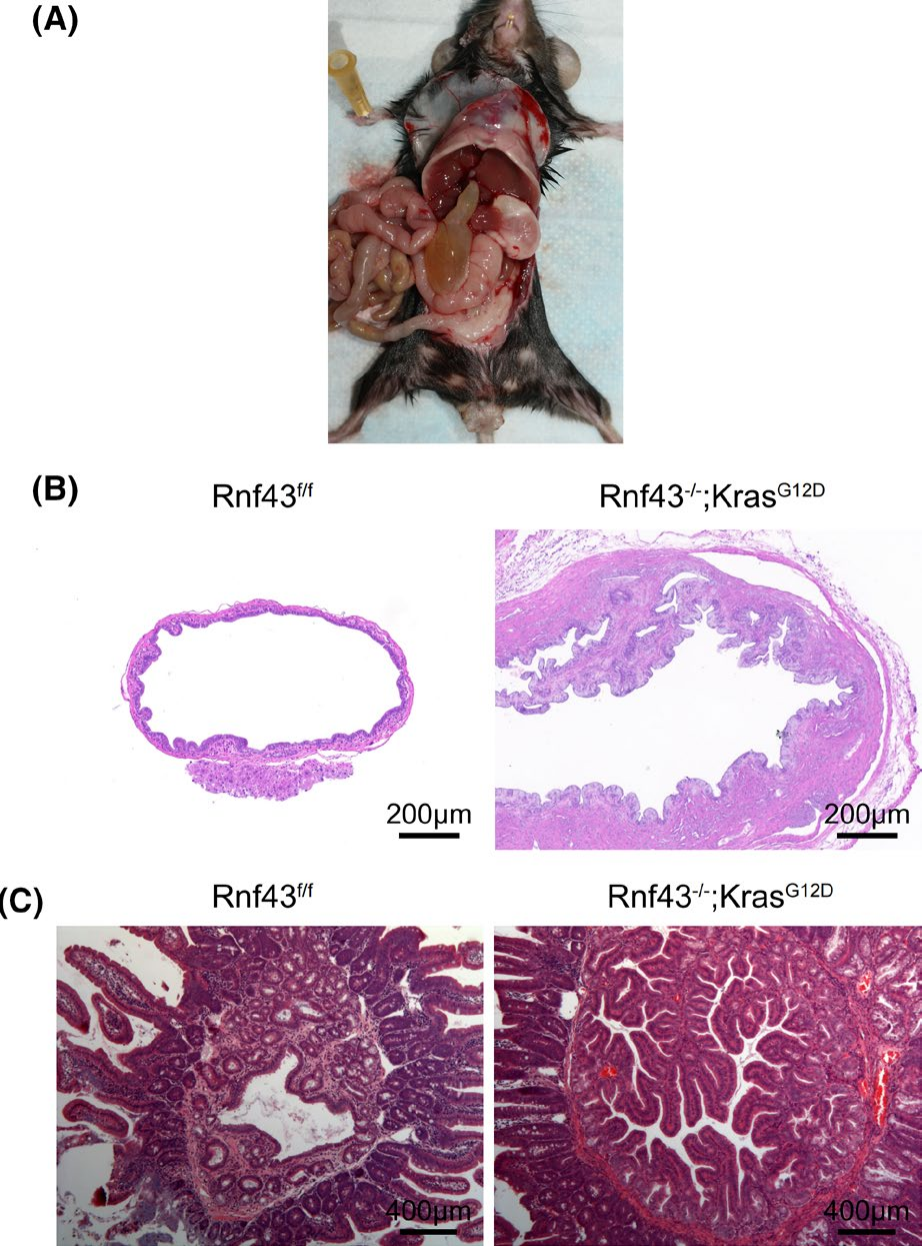
Biliary obstruction in pancreatic *Kras^G12D^* and *Rnf43* deficient mice. A, A 6‐month‐old *Rnf43* ^−/−^; *Kras^G12D^* mouse with biliary obstruction and gallbladder distension. B, H&E staining of 6‐month‐old *Rnf43* ^−/−^; *Kras* ^G12D^ mouse and control mouse bile ducts. Scale bar, 200 μm. C, H&E staining of 6‐month‐old *Rnf43* ^−/−^; *Kras* ^G12D^ mouse and control mouse major duodenal papilla. Scale bar, 400 μm

Unlike *Kras^G12D^* mice which did not develop obvious lesions, 6‐month‐old *Rnf43* ^−/−^; *Kras^G12D^* mice harbored biliary obstruction and gallbladder distension (Figure 5A). Pathological analysis demonstrated hyperplasia in bile duct epithelia of *Rnf43* ^−/−^; *Kras^G12D^* mice (Figure 5B) and major duodenal papilla (Figure 5C). These manifestations are commonly seen and have devastating consequences in pancreatic cancer patients.

### 3.5 Mutation co‐occurrence of *RNF43* and *KRAS* in human pancreatic adenocarcinoma

**FIGURE 6:**
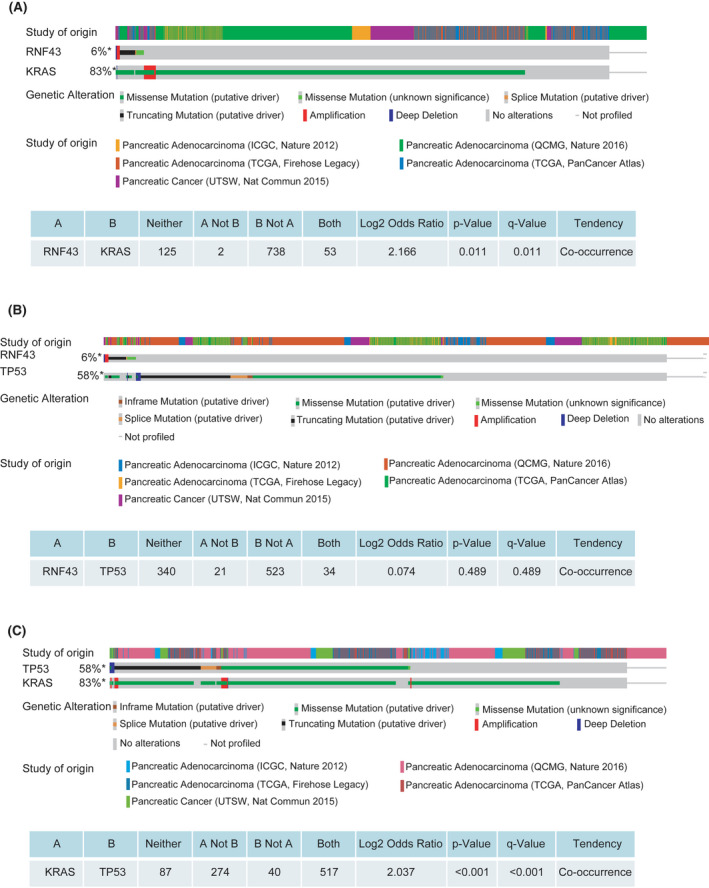
Concurrent mutations of *RNF43* and *KRAS* in human pancreatic adenocarcinoma. Landscape of *KRAS*, *TP53* and *RNF43* genetic alterations in pancreatic cancer deposited in ICGC, QCMG, UTSW and TCGA database of cBioPortal. A, Alteration co‐occurrence and mutual exclusivity between *RNF43* and *KRAS*. B, Alteration co‐occurrence and mutual exclusivity between *RNF43* and *TP53*. C, Alteration co‐occurrence and mutual exclusivity between *KRAS* and *TP53*

To determine the clinical relevance of our findings from mice, we analyzed the genomic data of 1034 pancreatic adenocarcinoma samples deposited in ICGC, QCMG, UTSW and TCGA databases with cBioPortal for *KRAS*, *TP53* and *RNF43* alterations. The most common aberration of *KRAS* is missense mutation. Missense mutation and truncating mutation are frequent in *TP53*. Truncating mutation and missense mutation are common in *RNF43*. The mutation frequencies of *KRAS*, *TP53*, and *RNF43* are 83%, 58%, and 6%, respectively. *KRAS* and *TP53* mutations occur together, while, in contrast, *RNF43* and *TP53* mutations do not co‐exist. Mutant *RNF43* almost exclusively occurs along with abnormal *KRAS* (Figure 6) and therefore, *KRAS* mutation is likely required for *RNF43* mutation‐associated pancreatic cancer development.

### 3.6 Intraductal papillary mucinous neoplasm development requires activation of Wnt/β‐catenin pathway

**FIGURE 7:**
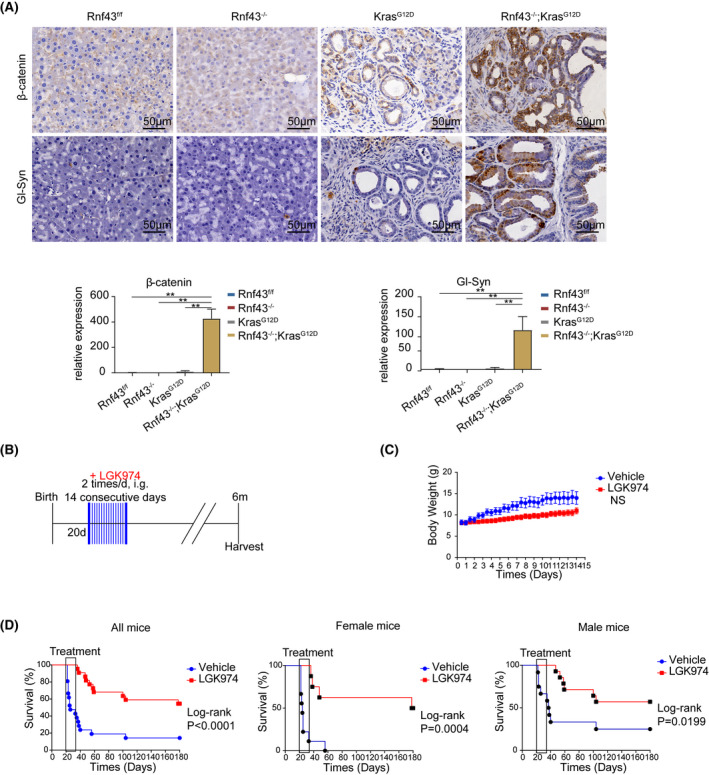
Wnt inhibitor blocks the development of pancreatic cystic neoplasms and prolongs the survival of the *Rnf43* ^−/−^; *Kras* ^G12D^ mice. A, IHC staining of pancreata from 6‐month‐old *Rnf43* ^f/f^, *Rnf43* ^−/−^, *Kras* ^G12D^ and *Rnf43* ^−/−^; *Kras* ^G12D^ mice (upper). Scale bar, 50 μm. Quantification of IHC stainings (lower). B, LGK974 treatment schedule. 20‐day‐old *Rnf43* ^−/−^; *Kras* ^G12D^ mice were divided into treatment group (LGK974, 22 in total, 14♂+8♀) and control group (Vehicle, 21 in total, 12♂+9♀). C, Body weights of LGK974‐treated and control mice during the treatment (means ±SEM). D, Kaplan‐Meier curves of *Rnf43* ^−/−^; *Kras* ^G12D^ mice with LGK974 (red) or vehicle (blue) treatment

β‐Catenin and its downstream target glutamine synthetase were highly expressed in the pancreata of 6‐month‐old *Rnf43* ^−/−^; *Kras^G12D^* mice with IPMN and PanIN (Figure 7A), suggesting that deficient *Rnf43* and mutated *Kras* activate Wnt/β‐catenin signaling. To investigate whether Wnt/β‐catenin signaling activation is indispensable to IPMN development, we treated *Rnf43* ^−/−^; *Kras^G12D^* mice with PORCN inhibitor LGK974 which blocks Wnt secretion. Because *Rnf43* ^−/−^; *Kras^G12D^* mice initiated pancreatic IPMN at 16 days and began to die by 21 days after birth, we chose 20‐day‐old *Rnf43* ^−/−^; *Kras^G12D^* mice for treatment. These mice were randomly assigned to experimental (LGK974) or control (MC/T‐80) groups. Mice were then administered with either MC/T‐80 or LGK974 in MC/T‐80 by oral gavage at a dosage of 5 mg/kg animal body weight, twice daily, for 14 consecutive days (Figure 6B). The body weight loss in LGK974 treated mice was not statistically significant (Figure 6C). While ~50% control mice died, none of LGK974‐treated mice died within the 14‐day treatment period (Figure 6D). After discontinuation of the treatment, mice from the experimental treatment group began to die, but higher mortality was still observed in sham‐treated mice than in LGK974‐treated mice (Figure 6D). The surviving mice were sacrificed at the age of 6 months. No gender difference was observed in response to the treatment (Figure 6D). Taken together, our data show that Wnt/β‐catenin signaling activation is necessary for pancreatic IPMN development and malignant transformation.

## 4 DISCUSSION

Loss‐of‐function mutation of *RNF43* coexists with gain‐of‐function mutation of *KRAS* in human IPMN and PDAC.^11^, ^12^, ^14^ To study the pathological effect of deficient *Rnf43* and active *Kras* on mouse pancreas, we first generated *Rnf43* ^−/−^; *Kras^G12D^* mice and then found that these mice developed cystic papillary lesions and invasive/metastasizing PDAC with aberrant activation of the Wnt/β‐catenin signaling pathway. Accordingly, PORCN inhibitor LGK974 blocked pancreatic tumor development and prolonged the survival of *Rnf43* ^−/−^; *Kras^G12D^* mice.

PDAC may arise covertly from noninvasive precursor lesions, including PCLs, after long latency.^3^, ^4^, ^5^, ^6^ Therefore, opportunities do exist for early detection and intervention of malignant transformation. Since the majority of PCLs are asymptomatic, and thus detected incidentally, and may never progress to advanced neoplasia, they do not need surgery or surveillance. We should thus stratify patients with distinct genetic alterations in their tumors and analyze their clinical features such as disease progression, treatment outcome, and survival. However, there are no reliable measurements to stratify PCLs for malignant potential. Consequently, almost all PCL patients have to undergo frequent surveillance with abdominal imaging and endoscopy which only benefits a small fraction of the patients. Even though pancreatectomy is technical challenge with significant morbidity and mortality, surgical resection is the only widely accepted treatment for IPMN with high‐grade dysplasia or invasive cancer.^7^, ^8^ However, more than 60% of resected mucinous PCLs are found to have low or intermediate dysplasia.^22^ Consequential overdiagnosis and overtreatment may have dire consequences, with significant risks of mortality and morbidity including exocrine and endocrine pancreatic insufficiencies.

Identification of the patients who do not require follow‐up and unnecessary surgery may help reduce the burden and societal costs for the majority of patients. To differentiate the cysts with malignant lesions from those without malignant potential, we need to dissect the molecular events underlying the malignant transformation. Although *RNF43* is the 3^rd^ most frequently mutated gene in pancreatic tumor tissues of IPMN patients, the causative relationship between loss of RNF43 and IPMN is yet to be established.^9^ To study the effect of RNF43 deficiency on pancreatic oncogenesis, we generated pancreatic *Rnf43* knockout mice. As deficient RNF43 caused bigger pancreata without obvious alterations of pancreas architecture, deficiency of RNF43 alone is not sufficient to cause pancreatic cysts. Mutant *KRAS* is thought to be the earliest driver gene in IPMNs,^21^ and mutant *RNF43* often coexists with mutant *KRAS*. These mutations are evolutionally selected in a subset of IPMNs.^10^, ^11^, ^12^ We observed development and progression of IPMN and PanIN in *Rnf43* ^−/−^; *Kras^G12D^* mice.

Concurrent activation of KRAS and GNAS simulated human IPMN lesions in mice.^23^, ^24^ However, GNAS does not accelerate KRAS‐mediated development of PDACs.^23^ In contrast, our *Rnf43* ^−/−^; *Kras^G12D^* mice developed invasive and metastatic PDAC much sooner than *Kras^G12D^* mice, resulting in reduced survival. Coexistence of mutated *RNF43* and *KRAS* in PDAC also supports a collaboration between RNF43 and KRAS in the development of human pancreatic cancer. Based on these findings, we suggest that close monitoring of lesion progression and more aggressive intervention such as surgery and novel therapeutics should be applied to IPMN with concomitant mutations of *KRAS* and *RNF43*.

Current management of pancreatic cysts is life‐long surveillance, endoscopic ultrasound‐guided fine‐needle aspiration, or surgery. No drugs are available for the treatment of pancreatic cysts. Single‐cell transcriptome sequencing of IPMN and PDAC revealed that many signaling pathways are altered, including Wnt/β‐catenin signaling, during progression from noninvasive dysplasia to invasive malignancy.^25^ We found that the Wnt/β‐catenin signaling pathway was activated in pancreatic *Kras^G12D^* and *Rnf43* null mice. RNF43 is one of the negative feedback regulators of the Wnt/β‐catenin signaling pathway.^17^, ^18^, ^19^ Oncogenic KRAS can also promote Wnt/β‐catenin signaling.^26^, ^27^, ^28^ Therefore active KRAS and deficient RNF43 collaborate to activate Wnt/β‐catenin signaling, even though the exact mechanism of how they synergistically activate this signaling cascade is yet to be dissected.

PORCN‐catalyzed palmitoylation of WNT is critical for WNT secretion into the cytoplasm.^29^ The PORCN inhibitor LGK974 selectively blocked the growth of RNF43 mutant PDAC cell line‐derived mouse xenografts.^19^ This inhibitor is now in a phase 1 clinical trial (NCT01351103) for patients with Wnt ligand‐dependent malignancies. We found that LGK974 reduced β‐catenin, abrogated pancreatic IPMN initiation and progression into PDAC, and dramatically prolonged the survival of *Rnf43* ^−/−^; *Kras^G12D^* mice without significant weight loss of the mice. Wnt/β‐catenin signaling activation is thus necessary for pancreatic IPMN initiation and development in *Rnf43* ^−/−^; *Kras^G12D^* mice. We suggest that a PORCN inhibitor would be effective and safe in the treatment of IPMN with *Rnf43* ^−/−^; *Kras^G12D^*.

In summary, Rnf43 is a tumor suppressor in the prevention of pancreatic precancerous lesions and PDAC development. Loss of RNF43 accelerates oncogenic KRAS‐driven IPMN and PDAC in mice. Activation of the Wnt/β‐catenin pathway as a result of the synergistic effect of RNF43 deficiency and KRAS^G12D^ promotes IPMN initiation and progression into PDAC, which can be abolished by LGK974. Mutated *KRAS* and *RNF43* detected from tumors, cyst fluid, pancreatic juice, and blood cell‐free DNA may serve as novel biomarkers of advanced neoplasia in the molecular surveillance and stratification for IPMN patients. Since the presence of KRAS and RNF43 mutations in IPMNs may predict future development of advanced neoplasia from PCLs, these PCLs should be closely monitored and treated with surgery and/or targeted therapeutics. Our findings may thus provide a window of opportunity and a rationale to prevent the progression of benign pancreatic tumors to malignant ones through surgery and/or medicine and thereby transform the care of a subset of the patients with PCLs. In addition, the biomimetic human disease model we have constructed should serve as a useful platform for further study of pancreatic tumorigenesis and drug screening.

## CONFLICTS OF INTEREST

The authors declare no conflict of interest.

## AUTHOR CONTRIBUTIONS

HZ, XZ, ZS, and JW conceived and designed the experiment. XZ, ZS, MZ, XQ, SY, LW, and LL performed the experiments and analyzed the data. YJ, WD, FL, and DJ assisted the study. JC analyzed the data. HZ, XZ, ZS and SY drafted and revised the manuscript.
